# Inertial Sensor-Based Assessment of Postural Control During Modified Romberg Conditions: Normative Reference Metrics from Healthy Adults

**DOI:** 10.3390/s26072093

**Published:** 2026-03-27

**Authors:** Mert Doğan, Nazmiye Erpan, Ceren Macuncu

**Affiliations:** Department of Physiotherapy and Rehabilitation, Faculty of Health Sciences, Akdeniz University, 07070 Antalya, Türkiye

**Keywords:** postural control, inertial measurement units (IMUs), modified romberg test, center of mass (CoM), sensory reweighting

## Abstract

Postural control relies on the integration of visual, vestibular, and somatosensory inputs under biomechanical constraints. Conventional Romberg testing provides limited quantitative insight, particularly regarding directional control and sensory dependence. Wearable inertial measurement units (IMUs) enable portable, multidimensional assessment of postural sway. Thirty healthy adults (15 females, 15 males) completed a modified Romberg protocol with systematic manipulation of stance (normal, tandem), visual condition (eyes open, eyes closed), and arm position (arms at sides, arms forward), including both left and right leading foot during tandem stance. Whole-body kinematics were recorded using a full-body IMU system comprising 17 wireless sensors. Center-of-mass (CoM) trajectories were derived from a 23-segment biomechanical model, and linear, spatial, and nonlinear sway metrics were computed. Statistical analyses were conducted using repeated-measures ANOVA, with significance set at *p* < 0.05. Visual deprivation significantly increased sway path length, mean sway velocity, and sway area across all stance conditions (*p* < 0.001). Tandem stance elicited greater mediolateral sway than normal stance (*p* < 0.001). Romberg ratios exceeded unity for all metrics and were significantly higher in tandem stance (*p* < 0.01). Arm position effects were negligible in normal stance but showed significant Vision × Arm interactions during tandem stance (*p* < 0.05). Leading foot position had no significant main effects. Combining a modified Romberg protocol with full-body IMU-based CoM analysis enables sensitive characterization of sensory dependence and directional postural control. Tandem stance with visual deprivation increases mediolateral postural demands under reduced base-of-support conditions, providing a more challenging context for evaluating directional postural control.

## 1. Introduction

Postural control, the ability to maintain, achieve, or restore equilibrium during static and dynamic activities, is fundamental to human function and a critical indicator of neurological, musculoskeletal, and sensory system integrity [1]. Accurate assessment of postural stability informs clinical diagnosis, rehabilitation planning, and fall-risk screening across diverse populations, ranging from older adults to individuals with neurological disorders [2,3]. While laboratory-based force plates are widely regarded as the gold standard for quantifying postural sway through center of pressure (COP) displacement [4], they are expensive, require fixed infrastructure, and are often impractical for routine clinical screening or ecological assessment [5]. Conversely, conventional clinical observational tests, such as the Romberg or tandem gait assessments, are accessible but rely on subjective judgment and often lack the sensitivity required to detect subtle or early-stage balance impairments [6]. This methodological dichotomy highlights the critical need for objective, quantitative tools that can bridge the gap between laboratory precision and clinical practicality.

Inertial measurement units (IMUs)—compact devices integrating tri-axial accelerometers, gyroscopes, and magnetometers—have emerged as a promising alternative to traditional systems [7]. Unlike force plates which measure ground reaction forces, IMUs directly capture body segment accelerations and angular velocities, offering a portable and ecologically valid means to assess movement dynamics [8]. IMU-based assessments allow for the quantification of a wide range of postural sway metrics, including linear measures such as root-mean-square (RMS) amplitude, total path length, and mean velocity, typically resolved into anterior–posterior (AP) and mediolateral (ML) components [9,10,11]. Distinguishing between these directional components is clinically vital, as AP control is primarily governed by ankle strategies, whereas ML control relies more on hip mechanisms, with each direction showing differential sensitivity to specific pathologies and sensory manipulations [3,4]. Beyond traditional linear metrics, the integration of IMU data enables the computation of nonlinear complexity-based measures, such as sample entropy. These measures quantify the regularity and predictability of postural sway, reflecting the adaptability of the postural control system [12]. Higher entropy typically indicates irregular, complex sway patterns associated with flexible and adaptive control, while lower entropy suggests rigid, stereotyped strategies often observed in aging or pathology [12,13].

A growing body of literature has validated the use of IMUs, particularly commercial-grade systems like Xsens, for quantifying postural sway. Studies have demonstrated strong correlations between IMU-derived metrics and force plate references under various conditions, including modified Romberg protocols [5,14]. Furthermore, IMU-based metrics have shown promise in discriminating between fallers and non-fallers and detecting impairments in clinical populations with greater sensitivity than observational tests [6,11]. However, despite these advancements, the clinical implementation of IMU-based posturography faces barriers due to heterogeneity in sensor placement, signal processing, and outcome reporting [10,15].

Significantly, gaps remain regarding the normative characterization of these metrics in healthy populations. Existing reference datasets are often limited to specific sensor brands or demographic subgroups, hindering generalizability [11,16]. Moreover, there is inconsistency in the literature regarding which specific metrics—linear versus nonlinear—and which directional components (AP vs. ML) are most sensitive to distinct postural challenges, such as variations in stance width, arm position, and visual feedback [9,11]. Understanding these fundamental sensorimotor strategies in healthy adults is essential for establishing clinical benchmarks and interpreting pathological deviations.

Although IMU-based postural assessments have been increasingly reported, most studies have focused either on validation against force plate systems or on limited stance manipulations using single- or few-sensor configurations [5,14]. There remains a need for structured, condition-specific reference data derived from model-based whole-body center-of-mass estimation under systematically graded sensory and biomechanical constraints. In particular, directional control characteristics and their modulation across stance width, visual availability, and upper limb positioning have not been comprehensively examined within a unified experimental framework. Addressing this gap is essential for translating wearable sensing approaches into standardized and interpretable clinical balance assessment protocols. The present study addresses these gaps by implementing a full-body IMU-based sensing framework comprising 17 synchronized inertial measurement units to quantify center-of-mass dynamics during modified Romberg conditions. Beyond biomechanical characterization, the study demonstrates the application of a wearable, multi-sensor architecture for multidimensional stabilometric analysis under systematically manipulated sensory and biomechanical constraints. Specifically, we aim to: (1) provide normative reference values for a comprehensive set of IMU-derived postural metrics, including linear measures (RMS sway, path length, mean velocity, 95% confidence ellipse area) and complexity measures (sample entropy), resolved into AP and ML components; (2) identify IMU metrics that are most responsive to experimentally imposed postural challenges related to stance configuration (normal vs. tandem), arm position (arms by side vs. arms forward), and visual condition (eyes open vs. eyes closed).

## 2. Materials and Methods

### 2.1. Study Design and Participants

This descriptive, cross-sectional study was conducted at the Department of Physiotherapy and Rehabilitation, Faculty of Health Sciences, Akdeniz University. A total of 30 right dominant healthy adult volunteers aged between 18 and 45 years were recruited. Inclusion criteria were: (1) absence of any neurological, orthopedic, or vestibular disorder; (2) no limitation in walking or standing abilities; and (3) ability to provide written informed consent; (4) right dominance. Exclusion criteria included inability to stand independently or maintain tandem stance for 30 s, history of vertigo, or development of dizziness or discomfort during testing.

Given the exploratory and normative nature of the study, the sample size was considered adequate and consistent with previous IMU-based postural control studies. Foot dominance was recorded based on the preferred leg used to kick a ball and considered during tandem stance interpretation.

The study protocol was approved by the Akdeniz University Clinical Research Ethics Committee, and all procedures were conducted in accordance with the Declaration of Helsinki (TBAEK-1153). Written informed consent was obtained from all participants prior to participation.

### 2.2. Instrumentation

Postural stability data were collected using the Xsens MVN Awinda motion capture system (Movella Inc., Enschede, The Netherlands). The system consists of 17 IMUs, each incorporating tri-axial accelerometers, gyroscopes, and magnetometers. Sensors were positioned in accordance with the manufacturer-defined full-body sensor placement protocol for the MVN Awinda system. The 17 IMUs mounted on predefined body segments, including the head, sternum, pelvis, upper arms, forearms, hands, thighs, shanks, feet, and scapular region, using the manufacturer-provided sensor fixation accessories to ensure stable attachment and alignment with the biomechanical model. Participant-specific anthropometric measurements were entered to scale the subject-specific biomechanical model. Calibration was performed using a standardized N-pose followed by a walking calibration. Kinematic data were acquired at a sampling frequency of 60 Hz. All measurements were conducted in an indoor laboratory environment following the manufacturer’s guidelines.

### 2.3. Experimental Protocol

Participants completed a modified Romberg test protocol comprising eight experimental conditions designed to systematically manipulate base of support, visual input, and upper extremity position. Each condition was performed three times for 30 s under standardized instructions. The protocol included normal stance (feet shoulder-width apart and parallel) and tandem stance (heel-to-toe alignment). For both stance conditions, trials were performed with arms positioned either at the sides or extended forward at 90° of shoulder flexion, under eyes open (EO) and eyes closed (EC) conditions. For tandem stance, trials were conducted with both the left and right foot positioned anteriorly. The order of testing conditions was randomized across participants to minimize order and learning effects. A trained spotter stood close to participants to ensure safety without interfering with sensor signals. For each participant and condition, the mean value of the three valid trials was used for statistical analysis. Rest periods were provided between trials to prevent fatigue.

**Figure 1:**
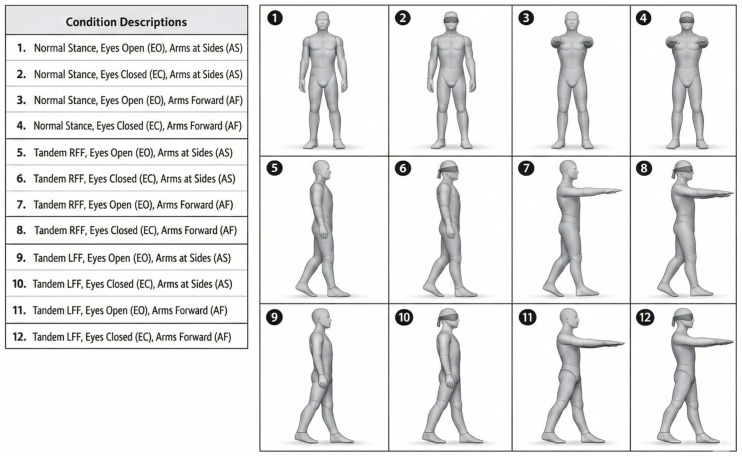
Schematic illustration of modified romberg conditions.

To enhance clarity of the experimental structure, a schematic illustration summarizing all stance, vision, arm position, and tandem foot variations has been added (Figure 1). The figure visually presents the full factorial design and the resulting 12 analyzed conditions, facilitating interpretation of the subsequent stabilometric comparisons.

### 2.4. Data Processing and Outcome Measures

#### 2.4.1. Signal Processing

Raw kinematic data were processed using MVN Analyze Pro 2025 software (Movella Inc., Enschede, The Netherlands) to estimate whole-body center of mass (CoM) trajectories based on a 23-segment articulated biomechanical model. Subsequent signal processing and metric computation were performed using custom-written scripts in Python (v3.9), employing NumPy (v1.21) and SciPy (v1.7) libraries. CoM displacement signals were detrended by removing the mean position to eliminate offset bias and to center the sway trajectories around the origin. Signals were then low-pass filtered using a zero-phase, fourth-order Butterworth filter with a cut-off frequency of 5 Hz to attenuate high-frequency noise while preserving physiologically relevant postural sway components. The laboratory reference frame was defined with the anterior–posterior (AP) direction aligned with the *X*-axis and the medio–lateral (ML) direction aligned with the *Y*-axis. To facilitate inter-individual comparisons, displacement-based measures were normalized to participant body height.

#### 2.4.2. Stabilometric Parameters

Postural stability was quantified using a core set of linear, spatial, and nonlinear stabilometric parameters derived from whole-body center of mass (CoM) displacement trajectories. Metrics were computed separately for the anterior–posterior (AP) and medio–lateral (ML) directions, as well as for resultant sway, in order to capture both directional and global characteristics of postural control [17].

Linear sway parameters were used to characterize the magnitude and temporal dynamics of postural motion. Sway path length (m) was defined as the total scalar length of the CoM trajectory over the trial duration and reflects the cumulative postural movement required to maintain equilibrium [4,17]. Mean sway velocity (m/s) was calculated by normalizing sway path length to trial duration and represents the rate of corrective postural adjustments and neuromuscular control demand [17,18].

Directional excursion and variability were quantified using range and root mean square (RMS) measures. Range AP and range ML (m) were computed as the difference between the maximum and minimum CoM displacement values along each axis, indicating peak-to-peak sway excursion. RMS AP and RMS ML (m) were calculated as the root mean square of CoM displacement relative to the mean position and provide robust estimates of sway variability that are less sensitive to extreme values than range-based measures. In addition, RMS resultant (m) was computed by combining AP and ML components, yielding a direction-independent measure of overall sway magnitude [1,4,17].

Spatial sway parameters were used to describe the two-dimensional area occupied by postural sway. The 95% confidence ellipse area (m^2^) was calculated using principal component analysis to enclose 95% of the CoM trajectory points in the horizontal plane, representing the spatial dispersion of sway. To capture boundary behavior and extreme excursions, the convex hull area (m^2^) was computed as the smallest convex polygon enclosing all CoM data points. To incorporate temporal information, sway area rate (m^2^/s) was derived by normalizing sway area measures to trial duration, reflecting the rate at which the postural control system explores its stability limits [19].

Nonlinear dynamics of postural control were assessed using sample entropy (SampEn), computed separately for AP and ML CoM displacement time series. SampEn quantifies the regularity of a time series by estimating the conditional probability that patterns of length m that match within a tolerance r remain similar at length m + 1 [5]. Lower SampEn values indicate more regular and predictable sway patterns, whereas higher values reflect greater complexity and adaptive variability. SampEn was calculated using an embedding dimension of m = 2 and a tolerance of r = 0.2 × SD, in accordance with established recommendations for postural sway analysis [20,21]. To facilitate interpretation, the Romberg ratio was calculated as the quotient of eyes-closed to eyes-open values (EC/EO) for each sway parameter and was used to quantify visual dependence [22].

### 2.5. Statistical Analysis

Statistical analyses were performed using Python (v3.9) with the SciPy (v1.7) and Pingouin (v0.5) libraries. Distribution normality for each stabilometric parameter was assessed using the Shapiro–Wilk test. A three-way repeated-measures analysis of variance (RM-ANOVA) was conducted with stance (normal, tandem), visual condition (eyes open, eyes closed), and arm position (arms at sides, arms forward) as within-subject factors. Sphericity was evaluated using Mauchly’s test, and Greenhouse–Geisser corrections were applied when the sphericity assumption was violated. Effect sizes were reported as partial eta squared (ηp^2^) for RM-ANOVA results and paired-sample Cohen’s d for post hoc comparisons. Statistical significance was set a priori at *p* < 0.05 for all analyses.

## 3. Results

A total of 30 healthy adults (15 males and 15 females) participated in the study. Participant demographic and anthropometric characteristics are summarized in Table 1. Recorded variables included age, body height, body weight, body mass index, foot length, shoulder height, shoulder width, elbow span, wrist span, arm span, hip height, hip width, knee height, and ankle height. All participants successfully completed the experimental protocol. No adverse events occurred during testing, and no trials were excluded due to balance loss, task non-compliance, or excessive movement artifacts. Mean age was comparable between males and females (22.26 ± 0.88 vs. 22.33 ± 0.82 years). Males exhibited greater body height (178.9 ± 7.2 cm vs. 166.8 ± 8.4 cm), body weight (74.1 ± 9.1 kg vs. 60.4 ± 7.2 kg), and body mass index (23.1 ± 2.2 kg/m^2^ vs. 21.2 ± 2.0 kg/m^2^). Segmental anthropometric differences were also observed, including foot length, upper limb spans, and lower extremity segment heights. Detailed descriptive statistics for all measured anthropometric variables are presented in Table 1.

Sway path length and mean velocity remained within narrow ranges across conditions, indicating relatively stable postural performance in this stance configuration. Directional measures demonstrated greater anterior–posterior excursion compared to mediolateral displacement. RMS values and resultant sway magnitude exhibited limited variability across experimental manipulations. Spatial parameters, including 95% ellipse area and convex hull area, remained low and demonstrated small condition-dependent fluctuations. Sway area rate values reflected modest CoM spatial exploration during normal stance. Nonlinear measures (sample entropy) showed minimal variation across visual and arm conditions in both anterior–posterior and mediolateral directions. Descriptive statistics for stabilometric parameters across normal stance position and additional conditions are presented in Table 2.

Inferential statistics examining the effects of normal stance, vision, and arm position on stabilometric parameters are presented in Table 3.

Sway Path Length was significantly influenced by both vision (*p* = 0.01, ηp^2^ = 0.23) and arm position (*p* = 0.02, ηp^2^ = 0.20), while Mean Sway Velocity showed similar significant main effects for Vision (*p* = 0.001, ηp^2^ = 0.27) and arm position (*p* = 0.02, ηp^2^ = 0.17). Range ML was significantly affected by arm position (*p* = 0.02, ηp^2^ = 0.20) but not Vision (*p* = 0.14), whereas Range AP showed no significant effects for either factor (*p* > 0.05). RMS ML was significantly altered by arm position (*p* = 0.02, ηp^2^ = 0.19) with no significant effect of Vision (*p* = 0.12), while RMS AP and RMS Resultant remained unaffected by the experimental conditions (*p* > 0.05). Sway Area Rate showed a significant main effect of arm position (*p* = 0.03, ηp^2^ = 0.16) but not vision (*p* = 0.18). No significant main effects were observed for 95% Ellipse Area (*p* > 0.05) or Convex Hull Area (*p* > 0.05). Structural complexity measures, Sample Entropy AP and ML, showed no significant main effects for vision or arm position (*p* > 0.05). Finally, no significant interaction effects between vision and arm position were observed for any of the postural sway parameters (*p* > 0.05).

In tandem stance, sway magnitude and velocity were markedly higher than in normal stance and increased substantially under visual deprivation (Table 4). This pattern was consistent for both left- and right-leading foot positions. Directional measures revealed greater mediolateral excursions compared to anteroposterior displacement, consistent with the reduced frontal plane base of support. RMS and resultant sway metrics demonstrated similar condition-dependent behavior. Spatial measures, including the 95% ellipse area, expanded under eyes-closed conditions, indicating increased center-of-mass dispersion during tandem stance. Leading foot position showed minimal descriptive differences relative to the effects of stance and vision. Descriptive statistics for stabilometric parameters across tandem stance position and additional conditions are presented in Table 4.

Inferential statistics examining the effects of tandem stance, vision, arm and foot position on stabilometric parameters are presented in Table 5.

Sway Path Length was significantly influenced by vision (*p* = 0.001, ηp^2^ = 0.34) but not by arm position (*p* > 0.05) or foot position (*p* > 0.05). Mean Sway Velocity similarly demonstrated a significant main effect of vision (*p* = 0.001, ηp^2^ = 0.36), whereas no significant main effects were observed for arm position or foot position (*p* > 0.05). Range AP showed a significant main effect of Vision (*p* = 0.01, ηp^2^ = 0.22) but was not significantly affected by arm position or foot position (*p* > 0.05). Range ML was significantly influenced by Vision (*p* = 0.001, ηp^2^ = 0.30), with no significant main effects of arm position or foot position (*p* > 0.05). RMS AP and RMS ML both demonstrated significant main effects of vision (*p* < 0.01, ηp^2^ ranging from 0.21 to 0.28), while arm position and foot position did not yield significant main effects (*p* > 0.05). RMS Resultant was also significantly affected by vision (*p* = 0.001, ηp^2^ = 0.31), with no significant contributions from arm position or foot position (*p* > 0.05) (Table 5).

For spatial sway measures, 95% Ellipse Area and Convex Hull Area exhibited significant main effects of vision (*p* < 0.01, ηp^2^ = 0.33 and 0.29, respectively), whereas arm position and foot position showed no significant main effects (*p* > 0.05). Sway Area Rate was significantly influenced by Vision (*p* = 0.002, ηp^2^ = 0.27), with no significant effects of arm position or foot position (*p* > 0.05). Structural complexity measures, Sample Entropy AP and Sample Entropy ML, showed no significant main effects for vision, arm position, or foot position (*p* > 0.05). Finally, no significant two-way or three-way interaction effects among vision, arm position, and foot position were observed for any of the postural sway parameters (*p* > 0.05) (Table 5).

**Figure 2:**
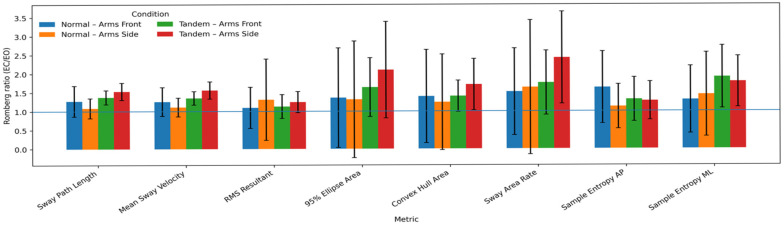
Romberg ratios of stabilometrics.

Descriptive statistics for Romberg ratios across all stabilometric parameters are illustrated in Figure 2.

Across all stabilometric parameters, Romberg ratios exceeded 1.0, indicating increased postural sway under eyes-closed conditions. The largest Romberg ratios were observed for sway path length, mean sway velocity, and RMS resultant, reflecting pronounced visual dependence in global sway magnitude measures. Spatial sway parameters, including 95% ellipse area, convex hull area, and sway area rate, similarly demonstrated elevated Romberg ratios, indicating increased spatial dispersion of postural sway following visual deprivation. In contrast, nonlinear complexity measures, sample entropy in the anterior–posterior and medio–lateral directions, exhibited comparatively smaller Romberg ratios, suggesting limited sensitivity of sway regularity to visual manipulation relative to magnitude-based metrics (Figure 2).

## 4. Discussion

This study examined postural control using a full-body IMU system under systematically manipulated sensory and biomechanical constraints. The findings underscore the hierarchical influence of sensory input and base-of-support configuration on balance regulation. Visual availability emerged as a dominant stabilizing factor, particularly when biomechanical stability was reduced. Narrowing the stance width modified directional control demands, indicating adaptive reorganization of postural strategies in response to mechanical constraints. While arm configuration demonstrated context-dependent modulation under higher task demands, leading foot position contributed minimally relative to sensory and stance-related factors. Collectively, these observations highlight the interaction between sensory integration processes and biomechanical boundary conditions in shaping center-of-mass control. These findings align with established models of sensory reweighting and demonstrate that IMU-based assessment can capture the multifaceted nature of postural control across clinically relevant conditions [23]. The observed patterns are consistent with previous validation studies showing that wearable inertial sensors provide valid measures of postural sway dynamics comparable to force plate assessments [10,24].

The marked influence of visual deprivation on postural stability is consistent with established evidence identifying vision as a primary contributor to upright balance regulation. Within the present experimental framework, the withdrawal of visual input amplified sway-related responses across stance conditions, indicating that visual information plays a stabilizing role even in young healthy adults. This finding aligns with previous sensor-based investigations demonstrating substantial modulation of postural sway under altered visual conditions [25]. From a sensorimotor perspective, the absence of visual feedback likely increases reliance on proprioceptive and vestibular inputs, thereby elevating corrective postural adjustments to maintain equilibrium

The elevation of Romberg indices under reduced visual conditions supports the interpretation of increased sensory dependence when biomechanical stability is challenged. The greater amplification observed in tandem stance suggests that narrowing the base of support intensifies reliance on visual input to maintain center-of-mass control. This pattern aligns with sensory reweighting theory, which posits that the central nervous system dynamically adjusts the relative contributions of sensory inputs based on their reliability and task demands [23]. Ferrari et al. reported that eyes closed to eyes open ratios of root mean square distance and 95% confidence interval ellipse area showed moderate to strong correlations (r = 0.65 to r = 0.87) between IMU and force plate measurements, supporting the validity of IMU-derived Romberg indices [10]. Martini et al. demonstrated that individuals with mild traumatic brain injury exhibited higher sensory reweighting scores on both firm (2.05 versus 1.38) and foam (3.09 versus 2.36) surfaces compared to controls, with closing eyes resulting in greater increases in sway area for the clinical group, highlighting the sensitivity of Romberg-type measures to impaired sensory integration [26].

The elevated Romberg ratios in tandem stance observed in the current study suggest that when biomechanical stability is compromised by a reduced base of support, the postural control system becomes more reliant on visual input to maintain equilibrium. This finding has potential clinical relevance, as it indicates that tandem stance with eyes closed imposes greater mediolateral and sensory demands than traditional Romberg testing in normal stance. Whether this translates into improved diagnostic sensitivity requires investigation in clinical populations.

Directional stabilization strategies appeared strongly dependent on stance configuration. Under wider base-of-support conditions, postural regulation predominantly engaged sagittal-plane control mechanisms, likely reflecting ankle-dominant strategies. In contrast, tandem stance imposed greater mediolateral stabilization demands, consistent with constrained frontal-plane support. This shift likely reflects increased reliance on hip and trunk contributions when mediolateral mechanical boundaries are reduced. Such directional modulation underscores the adaptability of the postural control system in response to biomechanical constraints. These directional patterns align with previous research documenting stance-dependent control strategies. Deshmukh et al. found that changes in stance configuration were more evident in the mediolateral direction, with thigh-mounted sensors showing a 336% increase in mediolateral sway during tandem stance, while ankle sensors were most sensitive to visual and surface manipulations in the anteroposterior direction [25]. Lee et al. reported that the mediolateral axis had higher complexity than other centers of pressure axes during single-leg stance with eyes open, and that all experimental actions were distinguishable in the forward-backward direction using force plate measurements [27].

The shift toward mediolateral-dominant control in tandem stance likely reflects a transition from predominantly ankle strategy in normal stance to increased reliance on hip strategy and lateral weight shifts when the base of support is constrained in the frontal plane. While the current study did not directly measure ankle versus hip strategy through segmental kinematics, the full-body IMU system provides the capability for such analyses in future investigations. The observed directional patterns are consistent with biomechanical models suggesting that narrow stance conditions necessitate greater hip and trunk involvement to control mediolateral stability [4]. The clinical relevance of these directional control patterns is substantial. Assessment protocols that include both normal and tandem stance conditions can reveal directional vulnerabilities that may not be apparent in standard clinical balance tests. Individuals with specific deficits in mediolateral control, such as those with vestibular disorders or hip weakness, may show disproportionate instability in tandem stance, providing diagnostic and intervention-planning information.

The effects of arm position on postural stability observed in this study were complex and context-dependent. In normal stance, arm position (arms at sides versus arms forward) produced minimal effects on sway measures, suggesting that when the base of support is adequate and postural demands are low, arm configuration does not substantially influence balance control. However, in tandem stance, arm position effects became more variable and condition-dependent, with significant Vision × Arm Position interactions indicating that the influence of arm configuration depends on the availability of visual input. The heterogeneity of arm position effects across individuals in tandem stance may reflect differences in individual postural control strategies, anthropometric characteristics, or prior motor experience. Some individuals may benefit from forward arm position through enhanced proprioceptive feedback or altered center of mass position, while others may find arms at sides more stable due to reduced moment of inertia or habitual movement patterns. This individual variability highlights the importance of personalized assessment and intervention approaches in clinical balance rehabilitation. Future research should systematically investigate arm position effects across a broader range of postural tasks and populations, including older adults and individuals with balance impairments. Understanding how arm configuration influences postural stability could inform clinical recommendations for optimal arm positioning during functional activities and balance training interventions.

This study demonstrates the methodological application of a full-body IMU sensing system for comprehensive center-of-mass–based postural analysis across multiple experimental conditions. The integration of synchronized multi-segment inertial data, signal filtering, and spatial–nonlinear stabilometric metrics illustrates a transferable sensing and processing framework for portable balance assessment systems. The Xsens MVN Awinda system enabled simultaneous measurement of multiple sway parameters, including path length, velocity, directional range and root mean square values, area measures, and derived indices such as Romberg ratios and directional ratios. This multi-parameter approach provides a more complete characterization of postural control than single-measure assessments and allows for detection of subtle changes across conditions.

The validity of IMU-based postural assessment has been established through numerous validation studies comparing wearable sensors to force plate measurements. Noamani et al. concluded that only accelerometer recordings are needed for monitoring standing balance dynamics, simplifying sensor requirements [24]. Ferrari et al. reported significant effects of eye conditions for almost all postural sway variables, with IMU-derived measures showing construct validity in healthy older adults [10]. Hansson et al. found strong coherence between IMU and force plate measurements, with coherence values of 0.84 for mediolateral sway with eyes open and 0.88 with eyes closed [28]. Neville et al. demonstrated that an inertial sensor showed significant differences between balance tests and high correlations with force plate (r = 0.793) and rigid-body kinematics (r = 0.887), with root mean square values increasing from 0.0368 in two-leg eyes-open stance to 0.911 in tandem eyes-closed stance on foam [29].

The clinical implications of this research are substantial. The modified Romberg protocol with systematic manipulation of stance, vision, and arm position provides a standardized yet comprehensive assessment framework that can be implemented in clinical settings using portable IMU technology. The elevated Romberg ratios in tandem stance suggest that this condition may provide enhanced sensitivity for detecting visual dependence and sensory integration deficits compared to traditional Romberg testing. The directional control patterns observed across stance conditions can inform targeted interventions addressing specific directional vulnerabilities.

IMU-based assessment offers several advantages over traditional force plate methods, including portability, lower cost, ease of use in diverse settings, and the potential for home-based or remote monitoring. Foulger et al. demonstrated that four IMUs are necessary to accurately estimate anteroposterior and mediolateral center of mass displacements across stance widths, with strong correlations (r = 0.92 ± 0.04 to 0.93 ± 0.03 for anteroposterior; r = 0.86 ± 0.13 to 0.97 ± 0.02 for mediolateral sway) [30]. These findings support the use of multi-sensor IMU systems for comprehensive postural assessment. In addition to many previous IMU-based balance studies that have employed single- or few-sensor configurations primarily for validation purposes or limited stance conditions, the present framework utilized a full-body inertial model to derive center-of-mass–based stabilometric metrics under systematically graded sensory and biomechanical constraints. This approach allows for a condition-specific characterization of postural control beyond simple sway quantification and positions the methodology within a model-based wearable posturography paradigm rather than a single-sensor validation design.

This study was conducted in healthy young adults, which may limit direct generalization to older or clinical populations with balance impairments. Postural sway was assessed using a single IMU-based system without concurrent force plate validation, and findings should therefore be interpreted within the context of this measurement approach. Additionally, the 30 s trial duration, while consistent with standard stabilometric protocols, may not fully capture longer-term postural adaptations or fatigue-related effects. Despite these considerations, the present study demonstrates several notable strengths. The systematic manipulation of visual input, stance configuration, and arm position within a single, standardized protocol enabled a comprehensive examination of postural control strategies under progressively increasing task demands. The 17-sensor configuration, while primarily used to improve the fidelity of whole-body kinematic tracking and center-of-mass estimation, also supports balance evaluation beyond laboratory constraints and enables portable assessments in non-laboratory settings. Furthermore, the portability of the wearable sensor–based protocol enhances its translational applicability in clinical, field-based, and future longitudinal research settings. Nevertheless, the requirement for multiple synchronized sensors may introduce cost and logistical considerations for routine clinical use, and simplified sensor configurations should be examined in future work.

This study highlights several important avenues for future research. First, extending the current assessment protocol to older adults and clinical populations with balance impairments would allow for the establishment of normative data. Incorporating concurrent force plate validation of IMU-based measures would enable direct comparison of measurement equivalence and strengthen the evidence base. Finally, longitudinal studies examining test–retest reliability and predictive validity for falls risk would consolidate the clinical utility of this approach. In parallel, future studies could directly compare full-body and reduced-sensor implementations to identify clinically feasible setups without substantial loss of measurement performance. Advanced analyses of IMU data (e.g., machine learning, frequency domain analysis) and comparisons in real-world conditions could offer significant contributions to personalized balance interventions. Additionally, future studies with larger samples should further investigate potential sex-specific influences on IMU-derived stabilometric parameters.

## 5. Conclusions

This study demonstrates that a full-body inertial measurement unit (IMU) system can sensitively quantify postural control adaptations under modified Romberg conditions. Visual deprivation emerged as the primary determinant of postural instability, while tandem stance increased postural demands and shifted control toward the mediolateral direction. Romberg ratios in tandem stance reflect increased visual dependence under reduced base-of-support conditions; however, these findings represent task-dependent modulation of postural control rather than established diagnostic or evaluative sensitivity. Arm position effects were modest and task-dependent, and leading foot position had limited influence. These findings support the utility of wearable IMU systems for standardized and comprehensive balance assessment. These findings support the methodological utility of synchronized multi-sensor IMU systems for standardized and multidimensional balance assessment, highlighting their potential as portable sensing solutions within digital postural evaluation frameworks.

## Figures and Tables

**Table 1:** Anthropometric and general characteristics of the participants.

Variable	Male (n = 15)	Female (n = 15)	Total (n = 30)
**Age (years)**	22.26 ± 0.88	22.33 ± 0.82	22.30 ± 0.84
**Height (cm)**	178.9 ± 7.2	166.8 ± 8.4	172.9 ± 9.8
**Weight (kg)**	74.1 ± 9.1	60.4 ± 7.2	67.3 ± 10.7
**Body mass index (kg/m^2^)**	23.1 ± 2.2	21.2 ± 2.0	22.2 ± 2.3
**Foot length (cm)**	27.2 ± 1.2	25.4 ± 1.1	26.3 ± 1.5
**Shoulder height (cm)**	146.4 ± 6.1	136.8 ± 6.9	141.6 ± 8.0
**Shoulder width (cm)**	46.5 ± 1.9	43.3 ± 2.2	44.9 ± 2.6
**Elbow span (cm)**	78.9 ± 3.3	73.6 ± 3.7	76.3 ± 4.4
**Wrist span (cm)**	100.3 ± 4.2	93.8 ± 4.7	97.1 ± 5.5
**Arm span (cm)**	178.9 ± 7.2	166.8 ± 8.4	172.9 ± 9.8
**Hip height (cm)**	95.0 ± 3.9	88.7 ± 4.6	91.9 ± 5.3
**Hip width (cm)**	34.2 ± 1.5	32.0 ± 1.6	33.1 ± 1.9
**Knee height (cm)**	51.0 ± 2.1	47.8 ± 2.4	49.4 ± 2.7
**Ankle height (cm)**	7.0 ± 0.3	6.6 ± 0.3	6.8 ± 0.4

Note. Values are presented as mean ± standard deviation.

**Table 2:** Descriptive statistics of stabilometric parameters for Normal stance in different conditions.

Condition	EO–AS	EC–AS	EO–AF	EC–AF
**Sway Path Length (cm)**	10.37 ± 2.60	10.69 ± 2.72	8.58 ± 2.02	10.46 ± 2.77
**Mean Sway Velocity (cm/s)**	0.33 ± 0.08	0.35 ± 0.08	0.28 ± 0.06	0.34 ± 0.09
**Range AP (cm)**	1.83 ± 0.72	2.03 ± 1.05	1.79 ± 1.22	1.78 ± 0.69
**Range ML (cm)**	0.91 ± 0.54	0.67 ± 0.38	0.65 ± 0.37	0.62 ± 0.39
**RMS AP (cm)**	0.41 ± 0.15	0.51 ± 0.39	0.44 ± 0.37	0.40 ± 0.16
**RMS ML (cm)**	0.21 ± 0.17	0.15 ± 0.10	0.15 ± 0.10	0.13 ± 0.06
**RMS Resultant (cm)**	0.47 ± 0.20	0.54 ± 0.40	0.48 ± 0.37	0.43 ± 0.16
**95% Ellipse Area (cm^2^)**	1.58 ± 1.98	1.36 ± 1.83	1.11 ± 1.03	0.89 ± 0.67
**Convex Hull Area (cm^2^)**	1.09 ± 1.08	0.89 ± 1.03	0.73 ± 0.59	0.70 ± 0.56
**Sway Area Rate (cm^2^/s)**	0.14 ± 0.10	0.18 ± 0.20	0.13 ± 0.13	0.12 ± 0.08
**Sample Entropy AP**	0.13 ± 0.05	0.14 ± 0.06	0.12 ± 0.06	0.16 ± 0.06
**Sample Entropy ML**	0.16 ± 0.07	0.19 ± 0.09	0.20 ± 0.11	0.20 ± 0.09

Note. EO: Eyes open; EC: Eyes closed; AS: Arms side; AF: Arms front; RMS: Root Mean Square; AP: Anterio-posterior; ML: Medio-lateral.

**Table 3:** Effects of vision and arm position on postural sway metrics during normal stance.

Parameters	Vision (*p*)	Vision (ηp^2^)	Arm Position (*p*)	Arm Position (ηp^2^)	Vision × Arm (*p*)	Vision × Arm (ηp^2^)
**Sway Path Length (cm)**	0.01	0.23	0.02	0.20	0.09	0.10
**Mean Sway Velocity (cm/s)**	0.001	0.27	0.02	0.17	0.15	0.08
**Range AP (cm)**	0.28	0.04	0.39	0.03	0.47	0.02
**Range ML (cm)**	0.14	0.08	0.02	0.20	0.14	0.08
**RMS AP (cm)**	0.25	0.05	0.30	0.04	0.22	0.05
**RMS ML (cm)**	0.12	0.09	0.02	0.19	0.38	0.03
**RMS Resultant (cm)**	0.69	0.01	0.16	0.07	0.30	0.04
**95% Ellipse Area (cm^2^)**	0.56	0.01	0.05	0.13	0.86	0.001
**Convex Hull Area (cm^2^)**	0.62	0.01	0.05	0.13	0.73	0.001
**Sway Area Rate (cm^2^/s)**	0.18	0.06	0.03	0.16	0.44	0.02
**Sample Entropy AP**	0.08	0.11	0.70	0.01	0.07	0.12
**Sample Entropy ML**	0.29	0.04	0.13	0.08	0.40	0.03

Note. ηp^2^: Partial eta squared. Effect size; RMS: Root Mean Square; AP: Anterio-posterior; ML: Medio-lateral.

**Table 4:** Descriptive statistics of stabilometric parameters for Tandem stance in different conditions.

	Left Foot Forward				Right Foot Forward			
Parameters	EO–AS	EC–AS	EO–AF	EC–AF	EO–AS	EC–AS	EO–AF	EC–AF
**Sway Path Length (cm)**	36.57 ± 6.66	51.93 ± 12.61	37.28 ± 6.78	50.06 ± 10.83	34.16 ± 5.97	53.82 ± 9.58	36.92 ± 6.36	53.19 ± 10.71
**Mean Sway Velocity (cm/s)**	1.17 ± 0.22	1.69 ± 0.38	1.20 ± 0.22	1.60 ± 0.34	1.10 ± 0.18	1.77 ± 0.31	1.21 ± 0.22	1.69 ± 0.31
**Range AP (cm)**	8.27 ± 3.22	8.86 ± 3.62	8.73 ± 3.47	10.45 ± 3.99	7.20 ± 2.74	9.37 ± 3.21	8.67 ± 4.15	10.67 ± 4.08
**Range ML (cm)**	12.11 ± 3.92	13.09 ± 3.42	12.10 ± 4.35	12.91 ± 3.98	11.55 ± 2.99	12.91 ± 3.69	11.95 ± 3.62	12.52 ± 3.44
**RMS AP (cm)**	1.53 ± 0.75	1.80 ± 0.77	1.64 ± 0.65	2.02 ± 0.76	1.38 ± 0.62	1.85 ± 0.76	1.73 ± 0.97	2.18 ± 0.97
**RMS ML (cm)**	1.91 ± 0.79	2.27 ± 0.79	2.04 ± 0.86	2.04 ± 0.64	1.90 ± 0.55	2.08 ± 0.51	2.01 ± 0.82	2.07 ± 0.67
**RMS Resultant (cm)**	2.52 ± 0.93	2.94 ± 0.95	2.67 ± 0.92	2.92 ± 0.81	2.40 ± 0.62	2.85 ± 0.68	2.76 ± 0.98	3.07 ± 0.99
**95% Ellipse Area (cm^2^)**	32.23 ± 23.67	50.96 ± 24.84	30.55 ± 14.27	49.73 ± 27.28	29.32 ± 18.01	53.30 ± 32.75	37.84 ± 24.15	54.71 ± 31.20
**Convex Hull Area (cm^2^)**	38.30 ± 22.76	51.75 ± 25.57	37.18 ± 19.37	54.97 ± 28.64	35.87 ± 19.30	55.53 ± 24.55	44.89 ± 24.85	58.00 ± 29.09
**Sway Area Rate (cm^2^/s)**	1.77 ± 1.15	3.36 ± 1.52	1.82 ± 0.80	2.94 ± 1.34	1.51 ± 0.61	3.45 ± 1.67	2.02 ± 1.04	3.40 ± 1.65
**Sample Entropy AP**	0.05 ± 0.03	0.07 ± 0.04	0.05 ± 0.03	0.05 ± 0.03	0.05 ± 0.03	0.06 ± 0.04	0.05 ± 0.04	0.06 ± 0.04
**Sample Entropy ML**	0.06 ± 0.04	0.09 ± 0.05	0.06 ± 0.03	0.10 ± 0.05	0.05 ± 0.02	0.10 ± 0.04	0.06 ± 0.04	0.11 ± 0.05

Note. Values are presented as mean ± standard deviation; EO: Eyes open; EC: Eyes closed; AS: Arms side; AF: Arms front; RMS: Root Mean Square; AP: Anterio-posterior; ML: Medio-lateral.

**Table 5:** Effects of vision, arm position, and foot position on postural sway metrics during tandem stance.

Parameter	Vision (*p*)	Vision (ηp^2^)	Arm Position (*p*)	Arm Position (ηp^2^)	Foot Position (*p*)	Foot Position (ηp^2^)	Vision × Arm (*p*)	Vision × Arm (ηp^2^)	Vision × Foot (*p*)	Vision × Foot (ηp^2^)	Arm × Foot (*p*)	Arm × Foot (ηp^2^)	Vision × Arm × Foot (*p*)	Vision × Arm × Foot (ηp^2^)
**Sway Path Length (cm)**	0.001	0.41	0.04	0.15	0.62	0.01	0.28	0.04	0.19	0.06	0.47	0.02	0.73	0.01
**Mean Sway Velocity (cm/s)**	0.001	0.43	0.03	0.16	0.58	0.01	0.31	0.04	0.21	0.05	0.44	0.02	0.69	0.01
**Range AP (cm)**	0.09	0.10	0.27	0.04	0.48	0.02	0.52	0.02	0.34	0.03	0.61	0.01	0.80	0.01
**Range ML (cm)**	0.01	0.24	0.03	0.16	0.55	0.01	0.18	0.06	0.22	0.05	0.39	0.03	0.77	0.01
**RMS AP (cm)**	0.07	0.11	0.19	0.06	0.51	0.02	0.29	0.04	0.26	0.04	0.42	0.03	0.71	0.01
**RMS ML (cm)**	0.02	0.21	0.04	0.15	0.60	0.01	0.25	0.04	0.31	0.04	0.46	0.02	0.75	0.01
**RMS Resultant (cm)**	0.003	0.38	0.05	0.13	0.59	0.01	0.27	0.04	0.24	0.04	0.41	0.03	0.72	0.01
**95% Ellipse Area (cm^2^)**	0.002	0.39	0.06	0.12	0.66	0.01	0.33	0.03	0.29	0.04	0.48	0.02	0.81	0.01
**Convex Hull Area (cm^2^)**	0.002	0.37	0.06	0.12	0.64	0.01	0.35	0.03	0.27	0.04	0.45	0.02	0.79	0.01
**Sway Area Rate (cm^2^/s)**	0.004	0.32	0.04	0.15	0.57	0.01	0.30	0.04	0.23	0.05	0.43	0.02	0.74	0.01
**Sample Entropy AP**	0.11	0.08	0.62	0.01	0.71	0.01	0.19	0.06	0.36	0.03	0.58	0.01	0.88	0.01
**Sample Entropy ML**	0.14	0.08	0.48	0.02	0.69	0.01	0.21	0.05	0.39	0.03	0.61	0.01	0.84	0.01

Note. ηp^2^: Effect size; RMS: Root Mean Square; AP: Anterio-posterior; ML: Medio-lateral.

## Data Availability

The datasets generated and analyzed during the current study are not publicly available due to ethical and privacy considerations but are available from the corresponding author upon reasonable request.

## Abbreviations

The following abbreviations are used in this manuscript:

ANOVA	Analysis of Variance
AP	Anterior–Posterior
AS	Arms at Sides
AF	Arms Forward
CoM	Center of Mass
EC	Eyes Closed
EO	Eyes Open
IMU	Inertial Measurement Unit
ML	Medio–Lateral
RMS	Root Mean Square
RM-ANOVA	Repeated Measures Analysis of Variance
SampEn	Sample Entropy
ηp^2^	Partial Eta Squared
